# Going on a vision quest in the pandemic context: the linkage between a learning organization, internal marketing and burnout in Ophthalmology physicians

**DOI:** 10.22336/rjo.2020.63

**Published:** 2020

**Authors:** Iuliana-Raluca Gheorghe, Victor Lorin Purcărea, Consuela-Mădălina Gheorghe, Ovidiu Popa-Velea

**Affiliations:** * “Carol Davila” University of Medicine and Pharmacy, Department of Marketing and Medical Technology, Bucharest, Romania; ** “Carol Davila” University of Medicine and Pharmacy, Department of Medical Psychology, Bucharest, Romania

**Keywords:** health care services, ophthalmology services, learning organizations, internal marketing, pandemic

## Abstract

The Romanian health care system faced a long line of chaotic reformations that proved to be less patient oriented and more expensive, leading to an increased level of burnout in physicians. The phenomenon of burnout occurs more and more often in the pandemic context, even in ophthalmology services. Among psychological and occupational factors that may cause burnout, there are also organizational factors that may decrease the burnout levels. The aim of this paper was to bring more insight into the prevention of burnout in ophthalmology physicians by applying strategies related to the organizational factors, namely, learning organization and internal marketing, in sustainable manner. We elaborated the conceptual framework on a thorough literature review and concluded that a learning organization has a positive impact on the implementation of internal marketing practices, internal marketing practices decrease the level of burnout, if appropriately applied, as well as, a learning organization has the power to decrease the level of burnout in ophthalmology physicians.

## Introduction

The current Romanian health care system has been described as being “chaotic” [**1**] and less patient-centred [**2**]. Moreover, during the last decade, the Romanian health care system has been at the core of many reformations and is constantly being the subject of everyday challenges, as health care consumers increased their demands and raised their expectations in receiving a “personalized” treatment in a cost-effective manner [**3**]. 

In a dynamic context, characterized by limited financial resources, a lack in infrastructure as well as by a long history of inappropriate health care reforms, the Romanian health care physicians present the burnout syndrome, regardless of age or specialty [**3**,**4**], even in Ophthalmology. 

Further, research conducted on burnout in medical backgrounds encompasses a vast palette of causal factors related to psychological, organizational and occupational factors [**3**]. In Romania, most studies have concentrated on the psychological and occupational factors, as linked to burnout [**1**,**3**], while the organizational factors have seldom been investigated. Thus, the aim of this paper was to fill in this knowledge gap, as more insight is needed in preventing burnout in Romanian health care professionals by using organizational factors, with a special interest in Ophthalmology. 

## Key concepts

Burnout

Special attention has been given to the phenomenon of burnout, particularly in human services professions [**5**]. From a professional perspective, burnout represents a psychological syndrome that occurs in response to chronic interpersonal stressors on the job as well as to longer exposure to a job connected stressful experience [**6**]. More exactly, professional burnout in individuals who work with people, is a syndrome of emotional exhaustion, depersonalization, and reduced sense of personal accomplishment [**5**]. 

In health care, burnout might be a common phenomenon among physicians with rates ranging from 25% to 60% depending on the medical specialty [**4**,**7**]. Moreover, at individual level, the consequences of burnout in physicians are associated with poor health [**8**], alcohol and drug use [**9**], and an increased risk of suicidal ideation [**10**], all of which have as outcome an increased risk of low-quality life, empathy [**11**], and, further, low job performance [**12**]. 

Immediate action is required when the burnout syndrome is manifested among physicians and a solution to its decrease is to implement efficient and sustainable organizational strategies. So, we strongly believe that all organizational factors may decrease the burnout syndrome in physicians, even in Ophthalmology. The most commonly reported organizational oriented factors that brought value to employees are the Learning Organization (LO) [**13**] and Internal Marketing (IM). 

Learning organization

The Learning organization (LO) is defined as “a vision that could help organizations to cope with and lead to environmental change, by enforcing learning activities” [**14**]. Moreover, Huysman described a LO as “a form of organization that enables the learning of its members in such a way that it creates positive value outcomes, such as innovation, efficiency, better alignment with the environment and competitive advantage” [**15**], and, in addition “is one that creates structures and strategies that facilitate the learning of all its members” [**16**]. In other words, a LO should ideally have “an increased organizational capacity to learn” in fast changing background [**17**], such as the pandemic context. Consequently, the crucial characteristics of a LO is adaptability, through continuous learning [**18**], and becoming a teaching organization [**19**], in fact, suggesting that the continuous learning process should “engage everyone into exploration, exploitation, and transfer of knowledge”, so as to increase the transformation of collective learning and also offer support in troubled times. 

Even if a clear definition of LO remains elusive [**19**], a number of particularities of an organization becoming a LO seem to recur. These particularities may refer to the following: it supports the continuous learning at individual, team or group and organizational levels [**17**, **20**], the creation and distribution of knowledge and information [**19**], the capacity to adapt to rapid change [**17**], the ability to change organizational behavior [**19**], and the ability to continuously transform [**20**]. 

From a LO perspective, health care organizations should be knowledge-intensive institutions, that encourage and implement continuous learning, so as to improve problem-solving capabilities of employees and to ensure a timely response to health care consumers’ needs [**21**]. In this context, we may assume that assessing a LO status in Ophthalmology may improve organizational performance, deliver value to health care consumers, raise their satisfaction and deliver quality services as well as generate positive word-of-mouth and increase loyalty. 

Internal Marketing

IM has developed as a strategy to assess the external consumer satisfaction, and further, to increase the organization’s performance [**22**,**23**], employee satisfaction [**24**] and organizational commitment [**25**]. Accordingly, IM is “concerned with making available internal products (jobs) that satisfy the needs of vital internal market (employees), while satisfying the objectives of the organization” [**23**], and later, employees may become internal customers [**23**]. 

Despite the fact that IM was integrated in many fields, the concept continues to be the subject of many debates, being associated mainly with personnel management, such as motivation, organizational commitment, communication and empowerment [**24**]. As such, the convergence of IM practices and principles revolve around employee development [**26**,**27**]. Hence, IM may be integrated in activities related to employee improvement of daily tasks, new work methods, greater knowledge of external customers, as well as their needs, values, practices and policies of the organizations and improve their quality of life [**25**]. More exactly, employee development concentrates on the creation of a consumers’ culture of knowledge [**28**], and they must know “what” and “why” a certain task is conducted [**29**]. In the same vein, the employee training may help employees in achieving skills and sensitivity to consumers’ needs [**26**].

In spite of the evidence provided by the scientific literature, it is clear that only a few organizations implement IM practices [**30**], and in those in which it is developed, it turns out it is improperly applied or it is carried out only by a small number of professionals [**31**]. Obviously, this also applies to Ophthalmology organizations. The weak application of this concept may reside in the fact that there are issues with the concept assessment in different fields, such as health care. In health care services, and especially in Ophthalmology, IM primarily deals with the methods used by the organization in managing the development and educational training of professionals, with a specific interest in communication skills, reward systems and satisfaction with work [**31**]. The efficient and sustainable implementation of IM practices may encourage the transmission of vision and goals and it may strengthen the employees’ skills in nurture service-oriented behavior [**31**]. 

## Conceptual framework

Given the context of health care services, and Ophthalmology specialty, we elaborated the following framework and hypotheses:

1. The Learning Organization positively influences the Internal Marketing. 

2. The Internal Marketing decreases Burnout.

3. The Learning Organization decreases Burnout. 

**Fig. 1:**
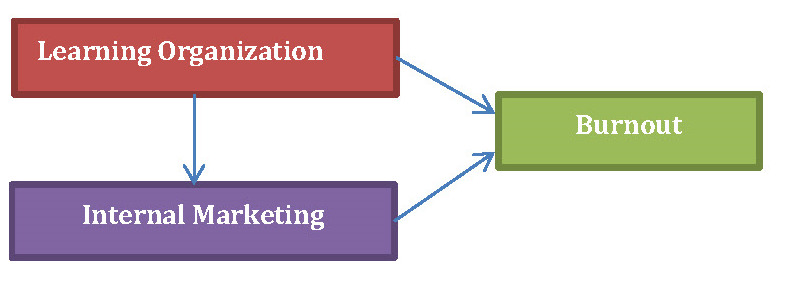
The conceptual framework

## Conclusion

Due to the rapid changes that take place in the pandemic context, health care organizations need to adjust their strategies and turn to organizational specific factors such as LO and IM in order to decrease the prevalence of burnout in Ophthalmology physicians. 

**Conflict of Interest**

The authors state no conflict of interest.

**Acknowledgements**

None.

**Sources of Funding**

None.

**Disclosures**

None.
